# Phenotypic characterization of NKT-like cells and evaluation of specifically related cytokines for the prediction of unexplained recurrent miscarriage

**DOI:** 10.1016/j.heliyon.2021.e08409

**Published:** 2021-11-16

**Authors:** Wafaa S. Khalaf, Mohammad R.A. Mahmoud, Walid F. Elkhatib, Hany R. Hashem, Wafaa E. Soliman

**Affiliations:** a Department of Microbiology and Immunology, Faculty of Pharmacy (Girls), Al-Azhar University, Nasr city, Cairo 11751, Egypt; b Department of Obstetrics and Gynaecology, Faculty of Medicine, Al-Azhar University, Nasr city, Cairo 11751, Egypt; c Department of Microbiology and Immunology, Faculty of Pharmacy, Ain Shams University, African Union Organization St., Abbassia, Cairo 11566, Egypt; d Department of Microbiology and Immunology, Faculty of Pharmacy, Galala University, New Galala city, Suez, Egypt; e Department of Microbiology and Immunology, Faculty of Pharmacy, Fayoum University, Al- Fayoum 63514, Egypt.; f Department of Microbiology and Immunology, Faculty of Pharmacy, Badr University in Cairo (BUC), Badr city, Cairo 11829, Egypt; g Microbiology and Immunology Department, Faculty of Pharmacy, Delta University for Science and Technology, Gamasa 11152, Mansoura, Egypt; h Biomedical Sciences Department, College of Clinical Pharmacy, King Faisal University, Al-Hofuf 36362, Al-Ahsa, Saudi Arabia

**Keywords:** NKT-like cells, Cytokines, Prediction, Unexplained recurrent miscarriage

## Abstract

**Problem:**

Immune system dysregulation is a major cause of unexplained recurrent miscarriage (URM). Women with URM need screening for their pregnancy microenvironment and *immune* regulators, to prevent spontaneous abortion.

**Method of study:**

In this study we evaluated NKT-like cell subsets in peripheral venous blood of women with URM using flow cytometry. The expression levels of specifically related Th1 cytokines (IFN-γ and IL-2), Th2 cytokine (IL-4), and Th17 cytokines (IL-17), were measured using enzyme-linked immunosorbent assay.

**Results:**

The percentage of CD16+CD56+NKT-like (Double Positive NKT-like; DPNKT-like) cell subset, and the levels of IL-2 and IFN-γ were significantly elevated in blood of non-pregnant and pregnant patients with URM compared with the healthy control groups, and these parameters were significantly increased after pregnancy in the same patients with URM. Based on the prevalence of the candidate immunological factors in patients with URM, the prognostic significance of the NKT-like cell subsets, IFN-γ and IL-2 profiles were evaluated as potential predictors of URM. A cut-off point of 2.55% for DPNKT-like cell subset in the blood and cut-off values of 39.5 and 20.5 pg/ml for the levels of IFN-γ and IL-2, respectively could be used for the prediction of the risk of spontaneous abortion. To the best of our knowledge, this is the first study that described the prognostic significance of the aforementioned immunological parameters before and after pregnancy, and highlighted the correlation of NKT-like cells and the candidate Th1 cytokines with pregnancy loss in women with URM.

**Conclusions:**

DPNKT-like cells, IFN-γ and IL-2 patient profiles could be used as markers to predict the risk of miscarriage in patients with URM.

## Highlights

• Disturbance in the immune system's regulators is an essential factor for recurrent miscarriage.
• There is an urgent need for women who have suffered pregnancy loss to identify specific immune regulators that underline recurrent miscarriage, to prevent and control miscarriage.
• CD16+CD56+NKT-like cells, Interferon-γ and Interleukin-2 patient profile could be utilized as biomarkers to predict risk of miscarriage in women who have suffered spontaneous miscarriage.

## Graphical abstract

**Figure:**
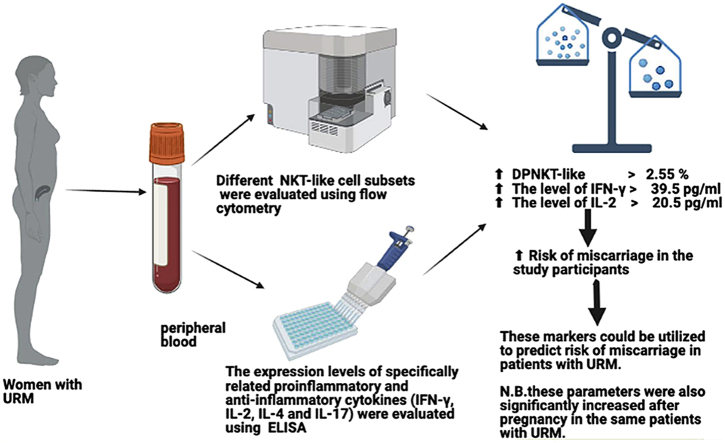

## 1 Introduction

Recurrent miscarriage is defined as two or more successive pregnancy losses within the first trimester [1]. Recurrent miscarriage affects about 15% of all pregnancies, and up to 50% of women who experienced recurrent abortions with no defined etiology [1]. The underlying etiology of recurrent miscarriage is highly debated, although there is considerable evidence suggesting a role for various immune system regulators in recurrent miscarriage [2]. However, the precise mechanisms by which the maternal immune system terminates the pregnancy remain poorly understood.

NKT-like cells have a major influence, controlling both the innate and the adaptive pregnancy immune responses [3, 4, 5, 6]. NKT-like cells were defined using the co-expression of CD3 and CD56 cell markers [7]. Unlike classical NKT cells, NKT-like cells are activated through the presentation of antigen in the context of human leukocyte antigen (HLA), however not via CD1d [8,9]. Vitelli-Avelar and colleagues further classified NKT-like cells with the additional inclusion of CD16 as CD3+CD56+CD16+ (DPNKT-like cells), CD3+CD56+CD16−, and CD3+CD56−CD16+ [11].

Elevated levels and activation of NKT-like cells in the blood have been reported to be accompanied with poor pregnancy outcomes [5, 6]. Decreases in CD3+CD56+ NKT-like cells following intravenous treatment with immunoglobulin has resulted in full-term pregnancy in women who suffered from recurrent miscarriage [10]. NKT cells activate many other leukocyte subsets, which may lead to a cascade of events, including trophoblast cell death [11, 12].

The function of NKT-like cells is influenced by the expression of CD16, which might lead to their ability to secrete specific pro-inflammatory cytokines including interferon-γ (IFN-γ), and interleukin-17 (IL-17) [13,14]. IL-17 promotes inflammatory processes, which can lead to the loss of pregnancy [15]. NKT-like cells can also regulate the balance of Th1/Th2 cytokines. This balance has a crucial impact on the regulation of embryo–maternal signaling [7, 16]. Subsets of T helper type 1 (Th1) cells secrete proinflammatory mediators such as IL-2, IFN-ɣ and tumor necrosis factor-alpha (TNF-α), which induce abortion [17]. However, subsets of T helper type 2 (Th2) cells typically produce anti-inflammatory mediators, including IL-4 and IL-10. These anti-inflammatory cytokines have immunosuppressive effects and play a vital role in the maintenance of pregnancy [18]. Throughout pregnancy, a bias toward Th2 cytokine production occurs, to suppress the production of Th1 cytokines. This shift to Th2 cytokines does not take place in women with recurrent abortion [19]. Activation of peripheral blood mononuclear cells (PBMCs) isolated from women during labor, using placental cells, leads to the secretion of Th2 mediators, while stimulation of PBMCs isolated from patients at spontaneous abortion caused the production of Th1 cytokines [17, 20].

NKT-like cells are therefore key candidates for molecules that can drive and modulate immunity during pregnancy [6, 12]. In the present study, the levels of different subsets of NKT-like cells and IFN-γ, IL-2, IL-4, and IL-17 cytokines were characterized and evaluated as potential immunological parameters that could be used for the prediction of pregnancy loss in women who had unexplained recurrent miscarriage (URM).

## 2 Materials and methods

### 2.1 Patients

The current study was performed from January 2019 to May 2021 in Cairo, Egypt. Peripheral venous blood (PVB) samples were collected from 90 non-pregnant patients with URM, and 90 non-pregnant women with healthy pregnancy histories were recruited as a control group. From all non-pregnant participants, a gynecologist collected blood samples during the luteal phase of their ovarian cycle. Of the 90 patients with URM, 42 women became pregnant, two patients dropped out of the study, so we assessed 40 samples before and after pregnancy, and compared their results with those of another 40 pregnant women with normal pregnancy history. Thirty-eight of our patients had miscarriages, and two patients had successful pregnancies. The samples were collected at a booking visit, between weeks 7 and 10. All 90 patients with URM had at least two successive idiopathic pregnancy losses in the first trimester. The characteristics of the study participants are listed in Table 1.

**Table 1:** Age, Duration of menstrual cycle, Body Mass Index (BMI) Number of miscarriages, and parity of the study participants.

	Non-pregnant patients with URM n = 90	Non-pregnant healthy women n = 90	Pregnant patients with URM n = 40	Healthy pregnant women n = 40	P
Age	20–40 (29.5)	20–40 (29)	28–34 (31.5)	26–38 (33)	NS
Menstrual cycle, duration, day	27.3–28.4 (27.9)	27.1–28 (27.7)	27.9–28.3 (28.2)	27.4–28.9 (28.3)	NS
BMI, kg/m^2^	25.6–26.9 (25.9)	24.7–28.9 (27.1)	25.9–27.9 (26.2)	24.6–26.7 (25.9)	NS
No. of URM	2–7 (3.1)	–	2–5 (3.7)	-	
Parity	0–1 (0.1)	1–6 (2.8)	0–1 (0.2)	1–4 (2.3)	

Data are presented as range (mean).

Our inclusion criteria were regular menstruation and normal levels of anti-cardiolipin antibody, phospholipid antibody, antithrombin III, anti-nuclear antibody, lupus anticoagulant, protein C and S, homocysteine, anti-thyroid peroxidase, prolactin, thyroid stimulating hormones TSH and thyroid hormones. Exclusion criteria were: a history of polycystic ovary syndrome (PCOS), parental or fetal chromosomal abnormalities, history of pregnancy-related infections, chronic disease, or current use of medication that might cause immunological variation.

All the healthy women in the control group had at least one live healthy child, with no previous history of spontaneous abortion. The control and the patient samples were assessed in parallel.

### 2.2 Ethics statement

Informed written consent was obtained from all participants, and all experiments were conducted according to the ethical policies and procedures approved by the ethics committee of the Faculty of Medicine, Al-Azhar University (Sayed Galal hospital), Egypt (approval no., Gyn_301Med.).

### 2.3 Flow cytometric analysis

Phenotypic analysis of PVB cells was carried out using direct immunofluorescent staining with PC5 conjugated anti-CD3, PE-labeled anti-CD56, and FITC conjugated anti-CD16 monoclonal antibodies (Becton Dickinson, UK), to identify NKT-like cell subsets. Fluorescently labeled isotype antibodies were used as negative controls. FACS Canto flow cytometer (Becton Dickinson, UK) was used for cell analysis, and FACS Diva software (Becton Dickinson, UK) was used for data analysis.

### 2.4 Enzyme-linked immunosorbent assay

Serum samples were obtained from fresh blood samples from all participants, frozen, and stored for cytokine measurement. The concentration of IFN-ɣ, IL-2, IL-4, and IL-17 in the serum samples were assessed using Quantikine enzyme-linked immunosorbent assay (ELISA) kits (R&D Systems, Minneapolis, MN, USA), following the manufacturer's instructions. Patient and control samples were analyzed in duplicate, to minimize the effect of intra-assay variation and technical error.

### 2.5 Statistical analysis

The differences between different subsets of NKT-like cells and cytokines were analyzed using nonparametric Mann–Whitney U tests. Correlations between the assessed variables were analyzed using Pearson's correlation, and combined predictive values were assessed using multiple logistic regression analysis. Receiver operating characteristic (ROC) curves were plotted for the URM and control group, using GraphPad Prism version 8.0.0 for Windows, GraphPad Software, San Diego, California USA”. Differences were considered to be statistically significant when P <0.05.

## 3 Results

### 3.1 Association of different NKT-like cell subsets with URM

**Figure 1:**
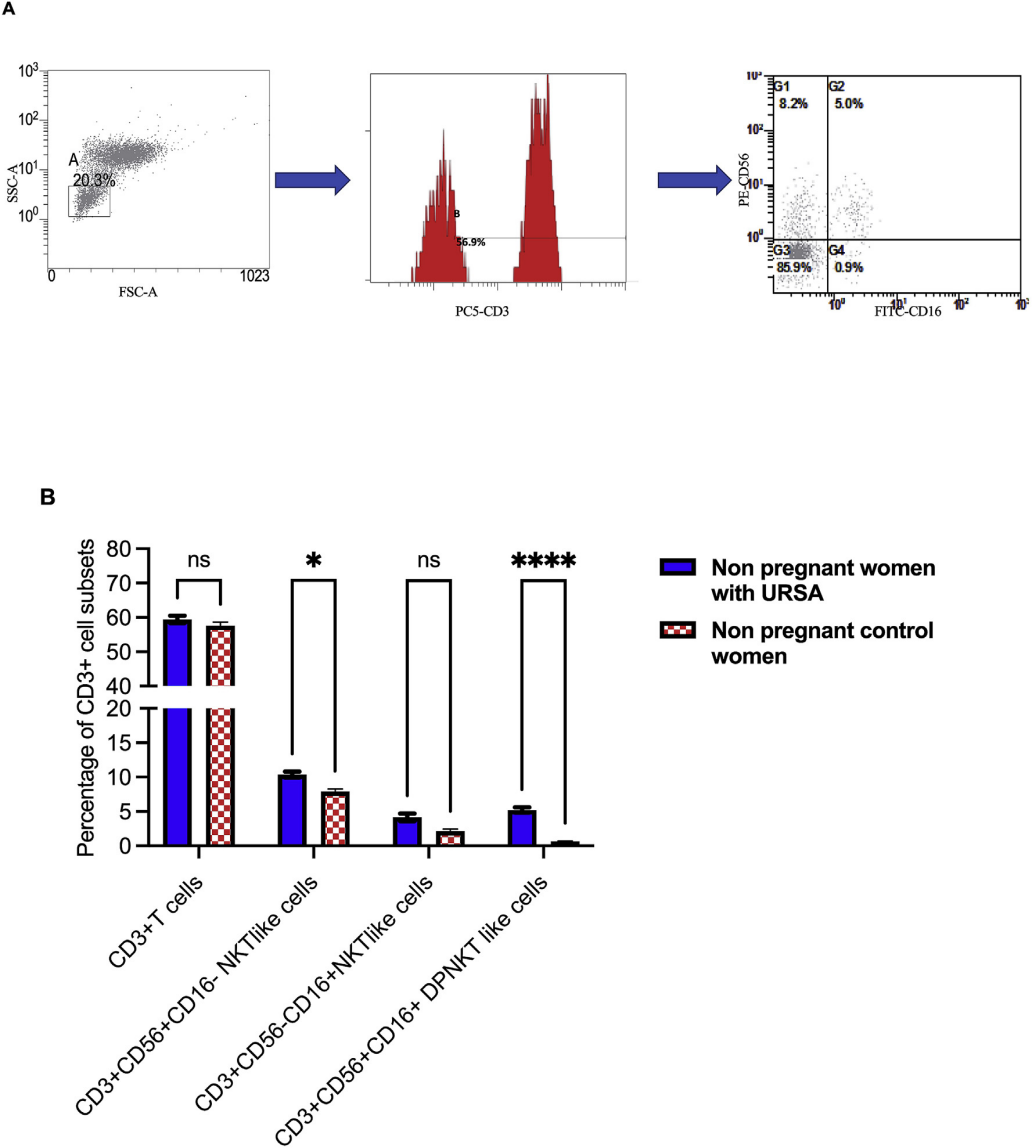
Percentage of CD3+T cell and different Natural Killer T cells (NKT)-like cell subsets in peripheral venous blood (PVB) of non-pregnant patients with unexplained recurrent miscarriage (URM). (A) Sequential gating on the lymphocytes using forward scatter X side scatter (FSCX SSC) dot plot, then gating the positive cells of CD3-PC5 histogram to represent CD3+ T cells, finally determining the percentage of different NKT-like cells subsets on the PE versus FITC dot plot. (B) Percentages of CD3+ T cell, CD56+CD16-NKT-like cells, CD56-CD16+NKT-like cells, CD56+CD16+DPNKT-like cells in PVB isolated from 90 non-pregnant women with URM history and 90 non-pregnant women with a normal history of pregnancy, followed by flowcytometric analysis. Results are presented as mean ± SE. The asterisks represent the significance level (∗ represent p <0.05, ∗∗ represent p ≤ 0.01, ∗∗∗ = P <0.001 and ∗∗∗∗ = P <0.0001).

To assess the percentage of CD3+T cells and different subsets of NKT-like cells (CD3+CD56+CD16+ (DPNKT-like cells), CD3+CD56+CD16−, and CD3+CD56−CD16+ NKT-like cells) in the PVB of URM patients and the healthy control group, flow cytometric analysis of these cell subsets was performed, using PVB samples isolated from 90 non-pregnant patients with URM and 90 healthy individuals (Figure 1A and B). The percentage of the CD3+T cell subset was not significantly different between non-pregnant patients with URM and healthy controls. The evaluation of different NKT-like cell subsets showed that the percentage of DPNKT-like cells subsets in non-pregnant women with a history of URM was significantly greater (P <0.0001) than that in non-pregnant women with a history of healthy pregnancy. The CD3+CD56+CD16-NKT-like cell subset was significantly elevated in non-pregnant patients with URM compared with the healthy controls (P = 0.0211). The percentage of the CD3+CD56-CD16+NKT-like cell subset was not significantly different among the tested groups (P = 0.0881).

**Figure 2:**
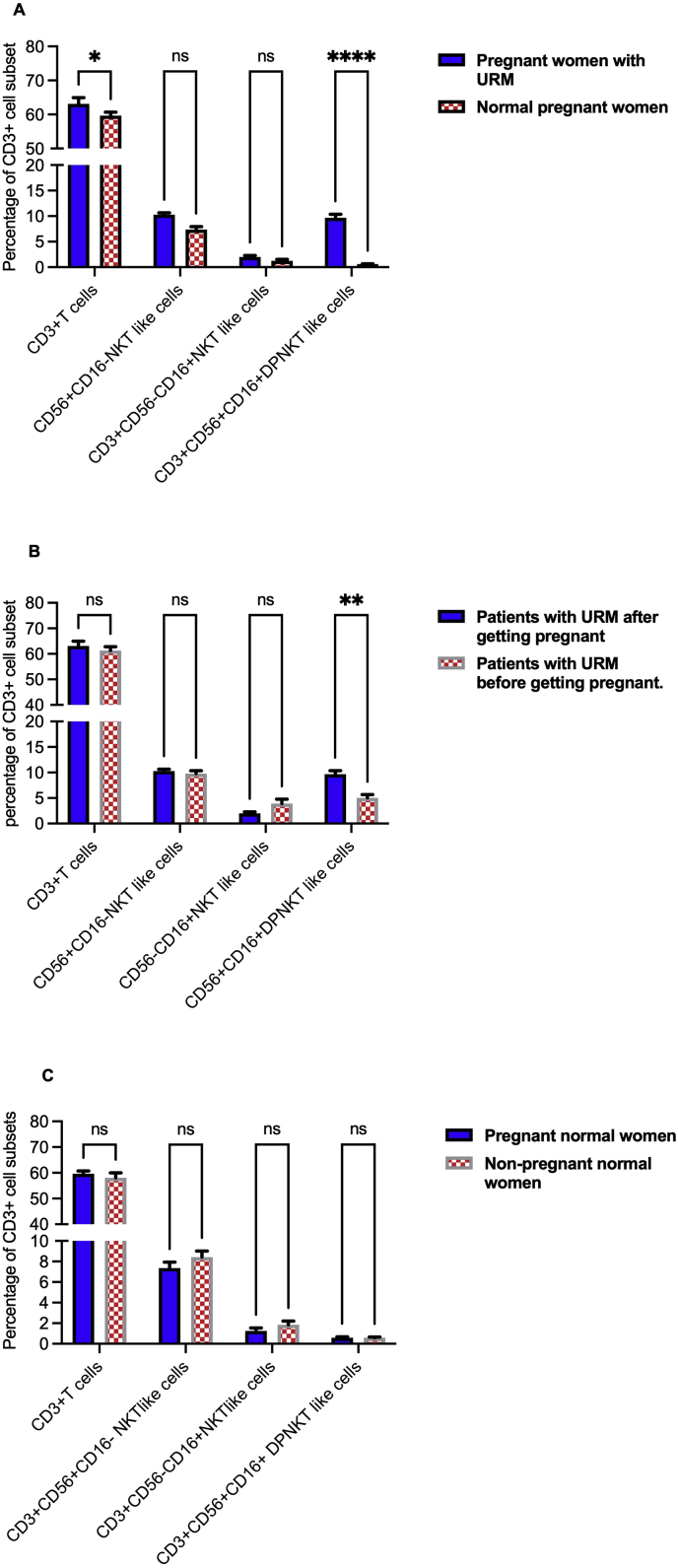
Percentage of CD3+T cell and different Natural Killer T (NKT)-like cell subsets in peripheral venous blood (PVB) of pregnant patients with unexplained recurrent miscarriage (URM). (A) Subsets of blood CD3+T cells and different NKT-like cell subsets in pregnant patients with a history of URM and women with healthy pregnancy history. (B) Percentage of CD3+T cell and different NKT-like cell subsets in patients with URM before and after they got pregnant. (C) Subsets of blood CD3+T cells and different NKT-like cell subsets in pregnant and non-pregnant women with normal pregnancy history. ∗ = p <0.05, ∗∗ = p ≤0.01, ∗∗∗ = P <0.001 and ∗∗∗∗ = P <0.0001.

Fourty of our URM patients became pregnant, providing an opportunity to compare the percentages of the candidate cell subsets before and after becoming pregnant, and with those with healthy pregnancies of the same gestational age. There was a significant (P = 0.015) elevation of CD3+T cells in pregnant patients with a history of URM relative to the healthy pregnant participants. In pregnant women with a history of URM, DPNKT-like cells were significantly elevated (P <0.0001) compared with control women of the same gestational age with a history of healthy pregnancy; and compared with their levels in the same women before becoming pregnant (P = 0.0038) (Figure 2A and B). The percentage of all candidate NKT-like cells subsets did not present any considerable difference between pregnant and non-pregnant women with healthy pregnancy history (P >0.05) (Figure 2C). These results support the potential involvement of DPNKT-like cells immunoregulators in the immune mechanism underlying recurrent failure of pregnancy.

### 3.2 Association of IFN-γ, IL-2, IL-4, and IL-17 cytokines and URM

**Figure 3:**
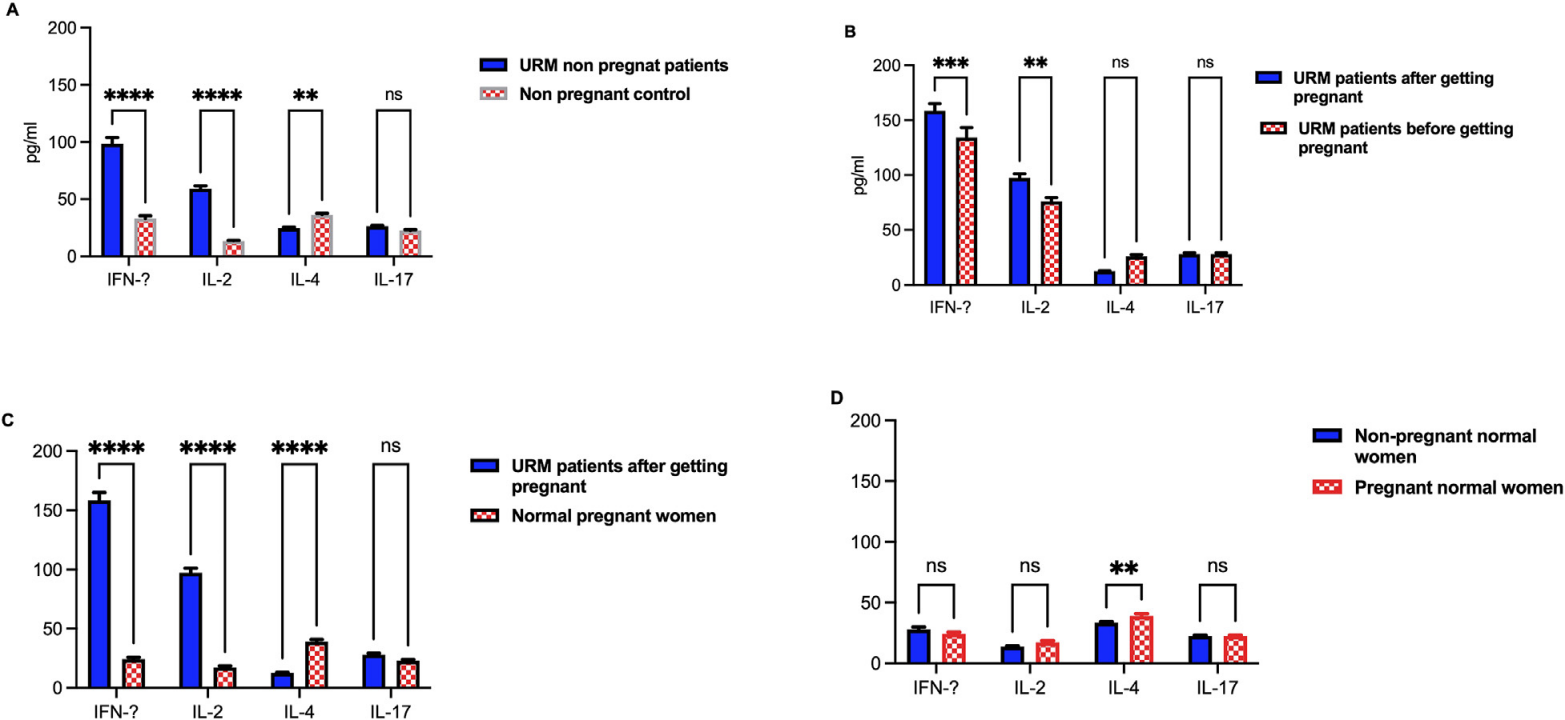
Cytokine expression level of Interferon-γ (IFN-ɣ), Interleukin-2 (IL-2), Interleukin-4 (IL-4) and Interleukin-17 (IL-17) in patients with URM. (A) The cytokine expression level of IFN-ɣ, IL-2, IL-4 and IL-17 in peripheral venous blood (PVB) isolated from 90 non-pregnant women with URM history and 90 non-pregnant women with a normal history of pregnancy. (B) The cytokine expression level of IFN-ɣ, IL-2, IL-4 and IL-17 in fourty pregnant women with URM history and fourty women with normal pregnancy history. (C) The cytokine expression level of IFN-ɣ, IL-2, IL-4 and IL-17 in fourty URM patients before and after they got pregnant. (D) The cytokine expression level of IFN-ɣ, IL-2, IL-4 and IL-17 in pregnant and non-pregnant women with normal pregnancy history. Results are shown as mean ± SE, ∗P< 0.05, ∗∗P <0.01, ∗∗∗ = P <0.001 and ∗∗∗∗ = P <0.0001.

Following detection of the level of NKT-like cell subsets in the PVB of patients with URM, levels of specific proinflammatory and anti-inflammatory related cytokines, such as IFN-γ, IL-2, IL-4, and IL-17 were evaluated using ELISA, in the same patients and control samples. In the non-pregnant patients with a history of URM, IFN-ɣ and IL-2 were significantly elevated (P <0.0001) compared with the healthy non-pregnant control group. Conversely, in non-pregnant women with URM the level of IL-4 was significantly lower (P = 0.0017), than that of the healthy control group (Figure 3A).

The levels of IFN-γ and IL-2 were significantly increased (P = 0.0005 and P = 0.0029, respectively) in pregnant patients with a history of URM compared with their levels before the pregnancy. The levels of IL-2 and IFN-ɣ were also significantly higher (P <0.0001) in pregnant patients with URM than in healthy pregnant women with the same gestational age. No significant decrease was found in levels of IL-4 in women with URM after becoming pregnant, compared with the levels before pregnancy. IL-4 was lower in pregnant patients with a history of URM compared with pregnant women with healthy pregnancy histories (P <0.0001). No significant difference was found in the level of IL-17 among any of the studied groups (Figure 3B and C). Also, no significant difference in the level of IL-2 and IFN-ɣ in PVB of pregnant and non-pregnant normal women. IL-4 was lower in non-pregnant normal women compared with pregnant normal women (P = 0.0078) (Figure 3D). Our results showed a consistent elevation of DPNKT-like cells percentage, and IL-2 and IFN-ɣ levels in the PVB of women with URM, indicating the importance of such immunoregulator and cytokine profiles in the prediction of spontaneous abortion.

### 3.3 Role of CD3+CD56+CD16+DP NKT-like cells, IL-2 and IFN-ɣ profile in the prognosis of URM

**Figure 4:**
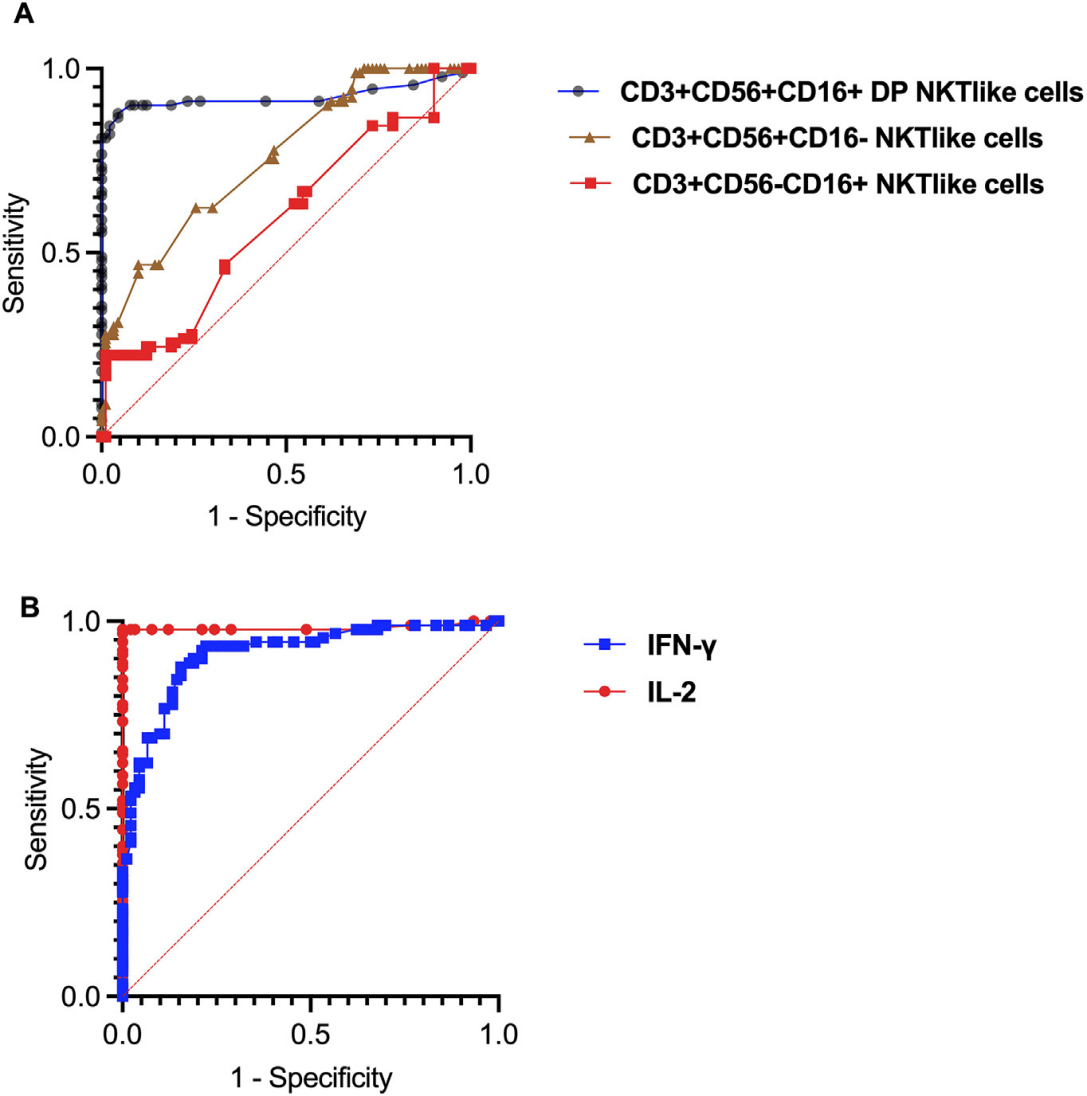
Receiver operating characteristic (ROC) curve for (A) different Natural Killer T cells (NKT)-like cell subsets. (B) Interleukin-2 (IL-2) and Interferon-γ (IFN-ɣ), for prediction of unexpected recurrent miscarriage.

Receiver operating characteristic (ROC) curves were constructed to evaluate the prognostic abilities of NKT-like cell subsets, IL-2 and IFN-ɣ in the prediction of URM, and to determine their optimal cut-off points. ROC curve analysis was performed for all non-pregnant women with (*n* = 90) and without (*n* = 90) URM. The DPNKT-like cells had the most appropriate cut-off point of 2.55%, and their area under the curve (AUC) was 0.9224. The area under the ROC curve for CD3+CD56+CD16-NKT-like cells was 0.7588 and the optimal cut-off point was 7.9% (Figure 4A).

At the cut-off point for DPNKT-like cells, the positive prognostic value (PPV) of having DPNKT-like cells above 2.55% was 97.4%. The negative prognostic value (NPV) of having this NKT-like cell subset equal to or below 2.55% was 86.2%. Using this cut-off point, the participants were divided into two groups. The first group represented women who had DPNKT-like cells higher than 2.55% (*n* = 78), with an incidence of recurrent abortion of 97.4%, while members of the second group (*n* = 102) had DPNKT-like cells of less than 2.55% and had a significantly lower incidence of recurrent miscarriage (13.7%).

We further investigated the prognostic accuracy of using IL-2 and IFN-ɣ in the prediction of URM. The analysis revealed that IL-2 with cut-off point of 20.5 pg/ml has an AUC of 0.982, a PPV of 97.7%, and a NPV of 97.7%. IFN-ɣ with a cut-off point of 39.5 pg/ml had an AUC of 0.9126 (PPV = 81.3% and NPV 91%). The incidence of URM in women with IL-2 higher than 20.5 was 97.7%, and with IFN-ɣ greater than 39.5 pg/ml was 81.3%. Women with IL-2 lower than 20.5 pg/ml and IFN-ɣ levels lower than 39.5 pg/ml had significantly decreased incidences of URM (2.2% and 8.9%, respectively) (Figure 4B). The area under the ROC curve of each of the NKT-like cell subsets, IL-2 and IFN-γ, Cut-off Point, Sensitivity, Specificity, P-value and 95% confidence interval are shown in Table 2. Additionally, mixed modeling was performed using multiple logistic regression analysis to combine these factors. The results showed that the combined Negative predictive power is 97.83% and the combined Positive predictive power is 100%. Also, DPNKT-like cells showed the highest Estimate and Odds ratios (1.259 and 3.521 respectively) among the variables, which indicate its importance in the prediction of miscarriage”.

**Table 2:** Area under the curve (AUC), Cut-off Point, Sensitivity, Specificity, P-value and 95% confidence interval for different NKT-like cell subsets of cells, IL-2 and IFN-γ

Test Result Variable(s)	Area	Cut-off Point	Sensitivity	Specificity	Asymptotic Significance	95% Confidence Interval (CI)	
						Lower Bound	Upper Bound
CD3+CD56+CD16+DPNKT-like cells	0.922	2.55%	0.811	0.988	0.0001	0.8736	0.9712
CD3+CD56+CD16-NKT-like cells	0.758	7.9%	0.622	0.7	0.0001	0.69	0.827
IL-2	0.982	20.5 pg/ml	0.977	0.977	0.0001	0.957	1.00
IFN-γ	0.912	39.5 pg/ml	0.933	0.777	0.0001	0.8699	0.955

### 3.4 Correlation analysis of the percentages of DPNKT-like cell with the candidate cytokine expression levels

**Figure 5:**
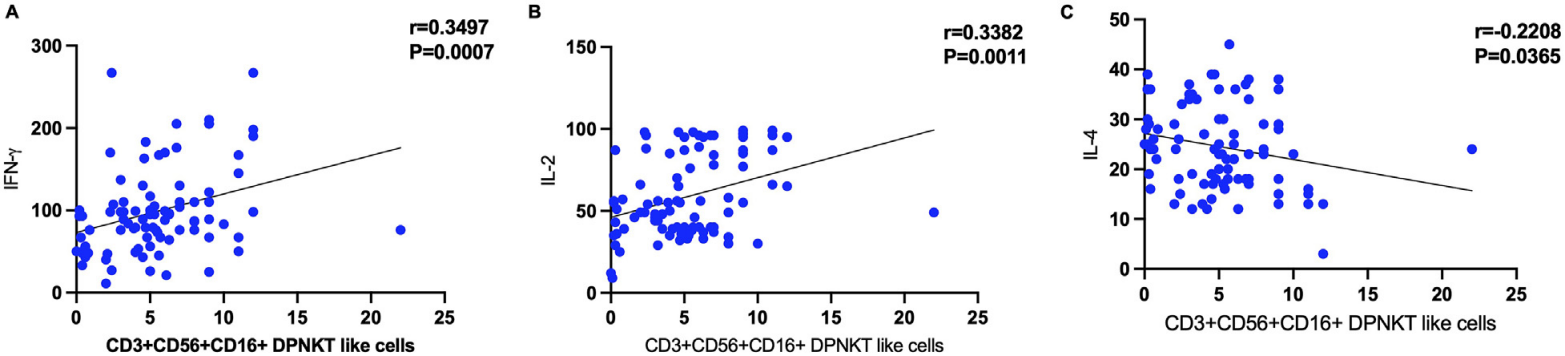
The correlation between the percentages of Double Positive NKT-like (DPNKT-like) cells and the expression levels Interferon-γ (IFN-ɣ), Interleukin-2 (IL-2) and IL-4.

Based on Pearson's correlation coefficients, the IFN-γ and IL-2 expression levels correlated positively with the percentage of DPNKT-like cells (r = 0.35, P = 0.0007) and (r = 0.34, P = 0.0011) respectively. However, there was a negative correlation (r = -0.22, P = 0.036) between IL-4 expression level and the percentages of DPNKT-like cells (Figure 5).

## 4 Discussion

There is an urgent need to identify specific immune regulators that underline recurrent miscarriage, so that women who have suffered pregnancy loss can be offered appropriate therapy to prevent and control miscarriage, even before getting pregnant. Disturbance of the regulators of the immune system, such as NKT cells, is an essential factor in recurrent miscarriage [21, 22]. The relationship between NKT-like cells and repeated miscarriage is one of the major areas of debate in reproductive immunology [5, 6]. In this study, we assessed the levels of different subsets of NKT-like cells and specific related Th1(IFN-γ, IL-2)/Th2 (IL-4) and Th17 (IL-17) cytokines, and their ability to predict recurrent miscarriage in women who have experienced URM, by comparing different case and control groups.

A unique aspect of this study is that we tested the same women with URM to track the changes in their pregnancy microenvironment before and during their first trimester of pregnancy, whereas most of the other studies selected only non-pregnant patients or women at specific stages of pregnancy. Additionally, the levels of NKT-like cells were directly measured from whole blood samples.

An appropriate amount of NKT cells is essential for pregnancy maintenance, as these cells are not present in the endometria of non-pregnant women [11], but the levels rise in early pregnancy [21, 22], and decrease in the last trimester of gestation [23]. These findings highlight the importance of NKT cells in immunotolerance during pregnancy. However, Abnormal levels of different subsets of NKT-like cells was observed in decidua and PVB isolated from patients with recurrent miscarriage, and following *in vitro* fertilization (IVF) in patients with implantation failure [5, 10, 24]. Our data showed significant elevation of DPNKT-like cells in the PVB of the non-pregnant women who had suffered spontaneous abortion, compared to the healthy non-pregnant group. The consistent elevation of such cell subsets and their role in recurrent miscarriage was verified by further increases in women with URM history after getting pregnant, and just before the incidence of miscarriage, relative to their levels in the same women with URM before pregnancy, and healthy pregnant women of the same gestational age. Accordingly, the presence of excessive amounts of NKT-like cells might affect the pathogenesis of unexplained recurrent abortion, these results go in agreement with Yuan et al [6]. There is limited and contradictory evidence that the NKT-like cells can affect recurrent miscarriage. Yamamoto et al showed that the percentage of NKT-like cells in the decidua of URM patients was less than that in healthy individuals [25]. These contradictory findings could be explained by the use of different sample sources (PVB vs decidua), and the time of the menstrual cycle at which samples were taken may affect the frequency of the immune regulators. No elevation was found in the level of CD3+ cells, except in pregnant women with URM compared with healthy pregnant participants. Such elevations in CD3+ T cells have previously been reported [26].

NKT-like cells can regulate the polarization of Th1, Th2, and Th17, and the secretion of Th1 proinflammatory cytokines such as IFN-ɣ and IL-2, and Th2 anti-inflammatory cytokines including IL-4 [7, 14, 16, 27, 28]. The NKT-like cells can also secret specific proinflammatory cytokines including IL-17 and IFN-ɣ, even without the need for clonal expansion [13, 14, 29]. IL-2 can also induce proliferation and activation of the NKT cells [29]. Thus, we hypothesize that the increased percentage of NKT-like cells may disrupt Th1/Th2 cytokine balance and promote inflammatory reaction that could lead to recurring miscarriage. Our data showed higher levels of IL-2 and IFN-γ in both non-pregnant and pregnant groups of women with URM, than in the non-pregnant normal control group and the control group of healthy women with the same gestational age. Further increased levels of IL-2 and IFN-γ in women who had experienced URM, after pregnancy and just before miscarriage in the first trimester of pregnancy, compared to their levels in the same patients before pregnancy, suggesting the harmful effect of these cytokines [30]. Lower level of IL-4 in both pregnant and non-pregnant groups of patients with URM, compared with the normal controls was also observed. Recurrent pregnancy loss in those patients may be explained by the proinflammatory effect of IL-2 and IFN-ɣ and the lack of anti-inflammatory effects of IL-4 (Th2 cytokines) [19]. Our results clearly indicate that the percentage of DPNKT-like cells was positively correlated with the levels of IFN-γ and IL-2 in PVB isolated from women with spontaneous abortion. While a negative correlation was found between the expression of IL-4 and the percentages of DPNKT-like cells. The expression of IL-4 was lower in non-pregnant compared with pregnant normal women, which support its role in normal pregnancy [18]. These results support TH2 bias in normal pregnancy and TH1 bias in women with URM, which may be caused by elevated levels of DPNKT-like cells that may lead to miscarriage in the early implantation stages [6, 30]. Importantly, to avoid any false results, we excluded URM patients with any pathological condition that might lead to any immunological changes, including elevation of IFN- γ.

Furthermore, we also noticed that the optimal cut-off values of 2.55% of DPNKT-like cells and increased the concentration of IFN-ɣ and IL-2 to 39.5 pg/ml and 20.5 pg/ml respectively, correlated negatively with pregnancy outcome, with good positive (97.4%, 81.3% and 97.7% respectively) and negative (86.2%, 91% and 97.7% respectively) predictive values. These results suggest the possibility of using DPNKT-like cells, IL-2 and IFN- γ levels as prognostic indicators of pregnancy outcome. Additionally, multiple logistic regression analysis showed respectable combined negative and positive predictive values (97.83% and 100% respectively). The DPNKT-like cells result agrees with the result published by Yuan et al (2015), with a higher cut-off point (3.75%). However, the sample size in their study was only 10 participants.

Thirty-eight of our patients lost their pregnancy in their first trimester, while two patients completed their pregnancy. In those two patients the levels of DPNKT like cells, IL-2 and IFN- γ were lower than the estimated cut off points, and even decreased after pregnancy, with slightly elevated level of IL-4. The successful pregnancy outcome could be attributed to the lack of the inflammatory effect of the candidate pro-inflammatory cytokines and the anti-inflammatory effect of IL-4 [17–20].

Some reports elucidated the role of IL-17 cytokines in pathological and normal pregnancy [31, 32, 33]. IL-17 is a proinflammatory mediator [14, 15] that stimulate the expression of many other proinflammatory cytokines [34]. IL-17 promotes inflammatory processes and conceptus antigens rejection, which can lead to the loss of pregnancy [15]. Our data point to non-significant difference in IL-17 levels in women experienced spontaneous abortion before and after pregnancy that supports the recent findings observed by Banja et al [33]. Elevation in IL-17 expression level was reported in women with recurrent miscarriage history [35]. Conversely, another study reported lower level of IL-17 in PVB of URM patients compared with PVB of fertile normal control women [33]. This contradictory data might be explained by the presence of different isoforms of IL-17 that may affect their function. In the current study, the used kit can evaluate IL-17A isoform explicitly. The correlation of IL-17F with recurrent abortion was reported previously [36]. An examination of different IL-17 isoforms and their relation to the URM is recommended in a future study.

The decidual specimen is the gold standard for the study of the uterine immune regulators but is unattainable for the prediction of miscarriage in pregnant women. Some limitations are associated with the use of such endometrium samples, including the invasive nature of endometrial sampling, and immune cell changes during various phases of menstruation, associated with hormonal changes [37]. Therefore, endometrial sampling was avoided in this study, and we used PVB, which eases the process of multiple sampling. Considering that the present study was designed to examine the changes in different subsets of NKT-like cells and specific cytokines in PVB isolated from women with URM.

## 5 Conclusion

In conclusion, our data support the hypothesis that high levels of NKT-like cells, IL-2 and IFN-γ are associated with an increased risk of URM. DPNKT-like cells, IL-2 and IFN-γ may therefore be used as biomarkers to predict miscarriage in women who have suffered spontaneous abortion.

## Declarations

### Author contribution statement

Wafaa S. Khalaf and Walid F. Elkhatib: Conceived and designed the experiments; Performed the experiments; Analyzed and interpreted the data; Wrote the paper.

Mohammad R.A. Mahmoud: Conceived and designed the experiments; Contributed reagents, materials, analysis tools or data; Wrote the paper.

Hany R. Hashem and Wafaa E. Soliman: Analyzed and interpreted the data; Contributed reagents, materials, analysis tools or data; Wrote the paper.

### Funding statement

This research did not receive any specific grant from funding agencies in the public, commercial, or not-for-profit sectors.

### Data availability statement

Data included in article/supplementary material/referenced in article.

### Declaration of interests statement

The authors declare no conflict of interest.

### Additional information

No additional information is available for this paper.
